# Supplementary material for: Differential influence of habit components on compulsive and problematic reward-seeking behavior
Source: PLOS Ment Health. 2025 May 21;2(5):e0000323. doi: 10.1371/journal.pmen.0000323 (PMC12798269; doi:10.1371/journal.pmen.0000323)
Supplement: S1 File — Psychometric properties of the French Creature of Habit Scale. (PDF) [file pmen.0000323.s001.pdf]

Supporting information for “Differential influence of habit components on compulsive and problematic reward-seeking behavior”

Lavinia Wuensch[1,2*], Yoann Stussi[1,2], Théo Vernede[2], Ryan J. Murray[2], David Sander[1,2], Julie Péron[1,3], Eva R. Pool[1,2]

1 Faculty of Psychology and Educational Sciences, University of Geneva, Geneva, Switzerland
2 Swiss Center for Affective Sciences, University of Geneva, Geneva, Switzerland
3 Department of Neurology, Cognitive Neurology Unit, University Hospitals of Geneva, Geneva, Switzerland

* lavinia.wuensch@unige.ch

S1 File. French validation of the Creature of Habit Scale

We verified that the French version of the COHS reproduced the two-factor structure of the original version of the COHS, distinguishing automaticity and routine components [1], using a psychometric network perspective [5] on the samples from Experiments 1 and 2. The sample consisted of 681 participants ($mean\ age = 24.11 \pm 7.83$; 506 women, 169 men, 6 non-binary). For this analysis, we used the R packages *psyTools* and *EGAnet* [6].

Please note that a French validation of the COHS already exists [2]. However, because 4 items were removed from that version [2], we attempted the current validation in which we were able to include the original 27 items with the same factorial structure as the original [1] and the German version [3] of the COHS.

Exploratory Graph Analysis

Dimensionality

To investigate the questionnaire dimensionality, we applied an exploratory graph analysis (EGA) with a Walktrap algorithm [4]. The analysis identified two dimensions in the questionnaire. The first dimension was composed of items associated with automaticity in the original questionnaire [1], while the second dimension was composed of items associated with routine in the original questionnaire (Fig 1A and Table 1). Fig 1 shows the standardized node strength for each dimension, which can be interpreted as the contribution of each item to the coherence of the automaticity and routine dimensions. The standardized node strengths suggest a clear structure without problematic cross-loadings, with item 18 and item 24 having the weakest loadings. Similarly to the original version of the questionnaire, the two dimensions were moderately correlated ($r = 0.25$, 95% CI $= [0.18, 0.32]$, $p < 0.001$)

Structural consistency

To compute the structural consistency, we applied a parametric EGA bootstrap procedure with 1000 iterations. The analysis revealed a stable structural consistency of

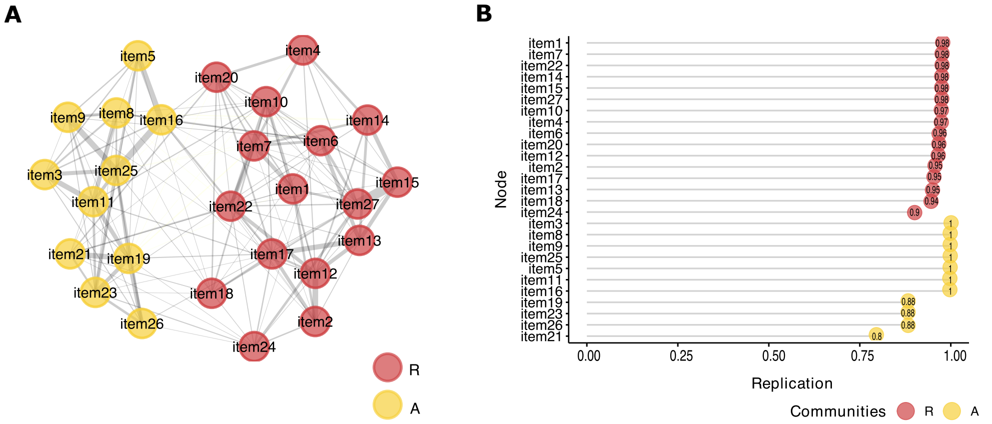

Fig 1. Depiction of the dimensions identified using exploratory graph analysis. A: The color of nodes represents dimensions and the thickness of lines represents the magnitude of partial correlations. B: Replication of each item in the two dimensions specified by exploratory graph analysis. A = automaticity dimension, R = routine dimension.

each dimension (85% for routine and 81% for automaticity), each individual item had a stability of at least 80% or higher (Fig 1B) with an average of 96 % for items of the routine dimension and of 95% for items of the automaticity dimension.

Subscale distributions

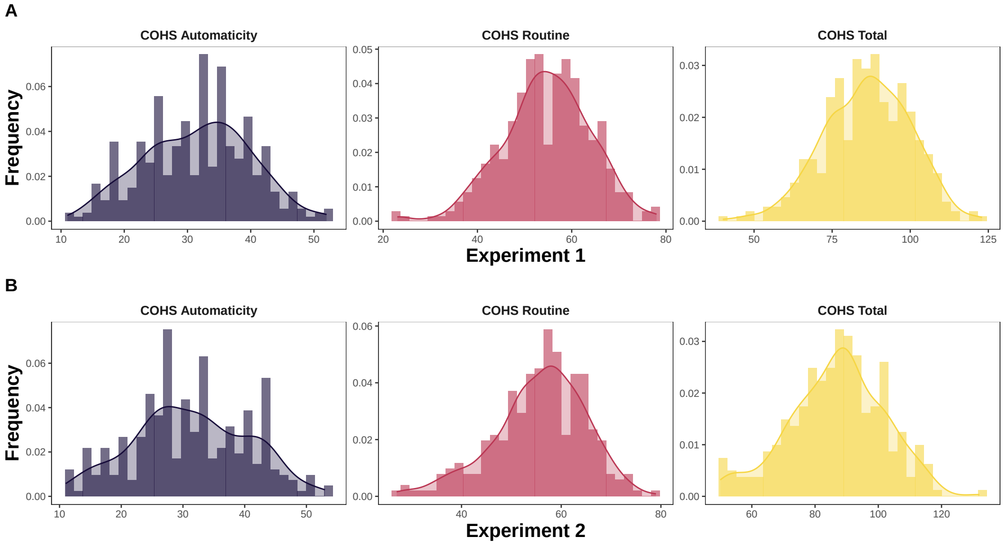

Fig 2. COHS and COHS subscale distributions in Experiments 1 and 2. A: Experiment 1. B: Experiment 2. COHS = Creature of Habit Scale, COHS Routine and COHS Automaticity = COHS Routine and Automaticity subscales.

Questionnaire items

Table 1. Standardized node strength for each node in each dimension identified by EGA.

	COHS Routine	COHS Automaticity
item 27	0.325	0.011
item 12	0.287	0.017
item 07	0.285	-0.026
item 15	0.279	< 0.0001
item 13	0.273	< 0.0001
item 17	0.219	0.047
item 22	0.212	0.025
item 01	0.196	0.008
item 10	0.188	0.039
item 02	0.185	0.014
item 14	0.182	-0.022
item 06	0.170	0.057
item 20	0.153	0.045
item 04	0.136	-0.003
item 24	0.089	0.041
item 18	0.084	0.024
item 25	-0.016	0.480
item 11	0.035	0.338
item 19	-0.043	0.337
item 03	< 0.0001	0.235
item 23	0.027	0.231
item 16	0.067	0.224
item 09	0.021	0.202
item 08	0.043	0.185
item 05	0.003	0.175
item 26	0.011	0.165
item 21	0.059	0.115

COHS Routine and COHS Automaticity = Creature of Habit Scale Routine and Automaticity subscales.

Table 2. French version of the Creature of Habit Scale.

Item	French text
01	J'aime bien garer ma voiture ou mon vélo toujours au même endroit.
02	Je cuisine d'habitude avec les mêmes épices et/ou arômes.
03	En passant devant une assiette de sucreries ou de biscuits, je ne peux pas m'empêcher d'en prendre un.
04	J'ai tendance à me coucher à peu près à la même heure tous les soirs.
05	Je prends souvent un snack quand je me déplace (par exemple, en conduisant, en marchant dans la rue, ou en surfant sur le web).
06	Je travaille volontiers dans ma zone de confort au lieu de me poser des défis, si ce n'est pas nécessaire.
07	J'ai tendance à faire les choses dans le même ordre chaque matin (ex., se lever, aller aux toilettes, boire un café. . .).
08	C'est typique de moi de manger des chips ou biscuits directement du paquet.
09	Chaque fois que je vais dans la cuisine, je regarde typiquement dans le réfrigérateur.
10	J'essaie toujours d'avoir la même place dans des endroits comme dans le bus, au cinéma, ou à l'église.
11	Je me retrouve souvent à finir un paquet de biscuits juste parce qu'il est là.
12	Normalement, j'achète la même nourriture au même supermarché.
13	Je compte sur ce qui a déjà été essayé au lieu d'explorer quelque chose de nouveau.
14	Je mange généralement les mêmes choses pour le petit déjeuner tous les jours.
15	J'ai tendance à aimer la routine.
16	Je me gâte habituellement avec un snack à la fin de la journée de travail.
17	Au restaurant, j'ai tendance à commander les plats que je connais bien.
18	Je fais partie de ces personnes qui sont vraiment énervées par les annulations de dernière minute.
19	Je me retrouve souvent à manger sans en être conscient-e.
20	Je m'assois habituellement à la même place à la table à manger.
21	Je me retrouve souvent à fonctionner en mode "pilote automatique", puis je me demande pourquoi je me suis retrouvé-e dans un un endroit particulier ou à faire quelque chose que je n'avais pas l'intention de faire.
22	Je suis toujours un certain ordre quand je prépare un repas.
23	La télévision me rend particulièrement enclin-e à manger de façon incontrôlée.
24	J'ai tendance à rester avec la même version de logiciel que je connais le plus longtemps possible.
25	Je me retrouve souvent à ouvrir le placard de la cuisine pour prendre un snack.
26	Je suis enclin-e à manger plus quand je me sens stressé-e.
27	Je trouve du réconfort dans la régularité.

References

1. Ersche KD, Lim TV, Ward LH, Robbins TW, Stochl J. Creature of Habit: A self-report measure of habitual routines and automatic tendencies in everyday life. Personality and Individual Differences. 2017;116:73–85. doi:10.1016/j.paid.2017.04.024.

2. Wyckmans F, Chatard A, Saeremans M, Kornreich C, Jaafari N, Fantini-Hauwel C, et al. Habitual routines and automatic tendencies differential roles in alcohol misuse among undergraduates. Frontiers in Psychology. 2020;11:607866. doi:10.3389/fpsyg.2020.607866.

3. Overmeyer R, Fürtjes S, Ersche KD, Ehrlich S, Endrass T. Self-regulation is negatively associated with habit tendencies: A validation of the German Creature of Habit Scale. Personality and Individual Differences. 2020;163:110029. doi:10.1016/j.paid.2020.110029.

4. Golino HF, Epskamp S. Exploratory graph analysis: A new approach for estimating the number of dimensions in psychological research. PloS one. 2017;12(6):e0174035. https://doi.org/10.1371/journal.pone.0174035

5. Christensen AP, Golino H, Silvia PJ. A psychometric network perspective on the validity and validation of personality trait questionnaires. European Journal of Personality. 2020;34(6):1095–1108. doi:10.1002/per.2.

6. Golino H, Christensen A. EGAnet: Exploratory Graph Analysis – A framework for estimating the number of dimensions in multivariate data using network psychometrics. R package version 2.1.1. 2023. 10.32614/CRAN.package.EGAnet.
