# Supplementary material for: Differential influence of habit components on compulsive and problematic reward-seeking behavior
Source: PLOS Ment Health. 2025 May 21;2(5):e0000323. doi: 10.1371/journal.pmen.0000323 (PMC12798269; doi:10.1371/journal.pmen.0000323)
Supplement: S2 File — Additional factor and network analyses on the restricted set of questionnaires used in both Experiments 1 and 2. (PDF) [file pmen.0000323.s002.pdf]

Supporting information for "Differential influence of habit components on compulsive and problematic reward-seeking behavior"

Lavinia Wuensch[1,2*], Yoann Stussi[1,2], Théo Vernede[2], Ryan J. Murray[2], David Sander[1,2], Julie Péron[1,3], Eva R. Pool[1,2]

1 Faculty of Psychology and Educational Sciences, University of Geneva, Geneva, Switzerland
2 Swiss Center for Affective Sciences, University of Geneva, Geneva, Switzerland
3 Department of Neurology, Cognitive Neurology Unit, University Hospitals of Geneva, Geneva, Switzerland

* lavinia.wuensch@unige.ch

S2 File. Replication

In order to further estimate the reliability of the links between habit and mental health problems, we ran additional factor and network analyses on the set of questionnaires used in both experiments: the COHS, CES-D, EAT, IAT, OCI, and PSS. The STAI-T and PMPUQ were therefore not included in the analyses for Experiment 1, and the EDS, GAS, LSAS, PCLS, QABB, SASSV, STICSA-T, and mYFAS were not included in the analyses for Experiment 2. Following the same data analysis strategy as described in the main text, we conducted an EFA followed by a network analysis for Experiments 1 and 2 each, allowing for a direct comparison between the factors and networks identified in each experiment.

Factor analysis

EFAs were performed on the 20 questionnaire subscales common to both Experiment 1 and 2 (Fig 1). The correlations between subscales were sufficiently large for an EFA (Experiment 1: $X^2_{(190)} = 3774.764$, $p < .001$, Experiment 2: $X^2_{(190)} = 1934.540$, $p < .001$). The factor analyses converged toward two very similar four-factor solutions (Fig 1).

The validity coefficients (Experiment 1: $R^2 = 0.966, 0.950, 0.963, 0.877$; Experiment 2: $R^2 = 0.965, 0.954, 0.900, 0.997$) assessing the potential impact of factor score indeterminacy [1] were satisfactory, allowing factor scores derived from the EFAs to be used in network analyses.

Network analysis

Fig 2 illustrates the dynamic networks we used to estimate the connections between the two components of habitual behavior, impulsivity, and the EFA-extracted mental health factors.

The resulting networks had strong similarities (Fig 2): routine and automaticity were positively related, automaticity was associated with problematic media while routine was associated with compulsivity, impulsivity was positively associated with

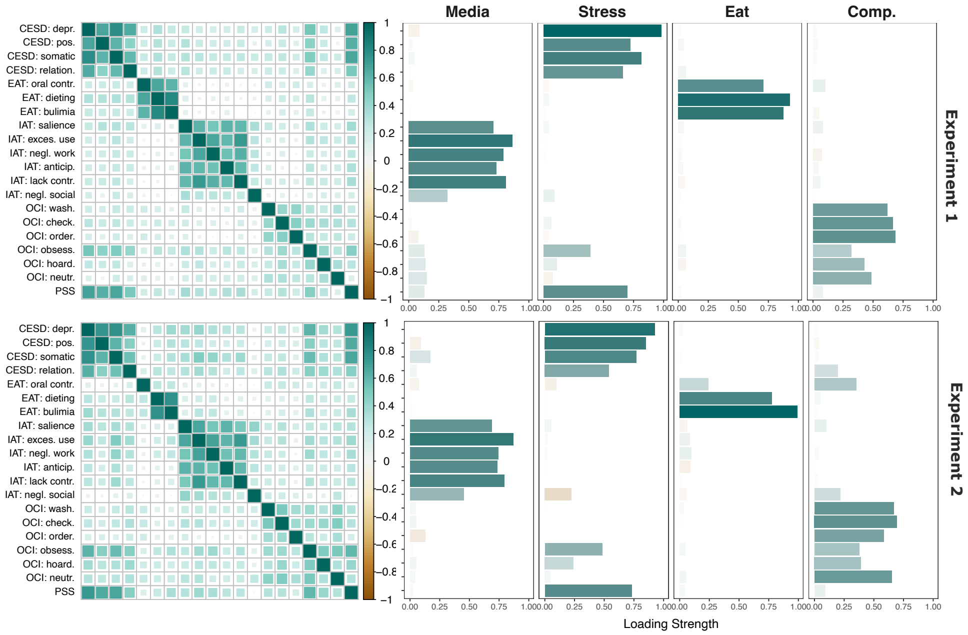

Fig 1. Correlation matrix and standardized loadings for Experiment 1 and 2, using only subscales common to both experiments. Standardized loadings for questionnaire subscales into each factor are displayed in green for positive values and yellow for negative values. CESD = Center for Epidemiologic Studies Depression Scale; EAT = Eating Attitudes Test; IAT = Internet Addiction Test; OCI = Obsessive-Compulsive Inventory Revised; PSS = Perceived Stress Scale; depr. = depressive affect; pos. = positive mood; relation. = disturbed interpersonal relationships; contr. = control; exces. use = excessive use; negl. = neglect; anticip. = anticipation; wash. = washing; check. = checking; order. = ordering; obsess. = obsessing; hoard. = hoarding; neutr. = neutralizing; danger. = dangerous use; prohib. = prohibited use; depend. = dependent use. $N_{experiment1} = 381$, $N_{experiment1} = 285$.

Table 1. Expected influence of the graphical LASSO network nodes, using only subscales common to Experiments 1 and 2.

	Experiment 1	Experiment 2
Automaticity	0.396	0.962
Routine	-1.049	-1.476
Problematic media	0.494	0.431
Stress	1.400	1.370
Problematic eating	-0.485	-0.768
Compulsivity	0.626	-0.024
Impulsivity	-1.382	-0.493

automaticity and negatively associated with routine, and affective stress was associated with all problematic behavior nodes.

The main difference between the two networks in terms of links between nodes was the positive association between automaticity and problematic eating in Experiment 2, which was not found in Experiment 1. Interestingly, while the overall network structures were similar, the centrality of each node within the network was variable

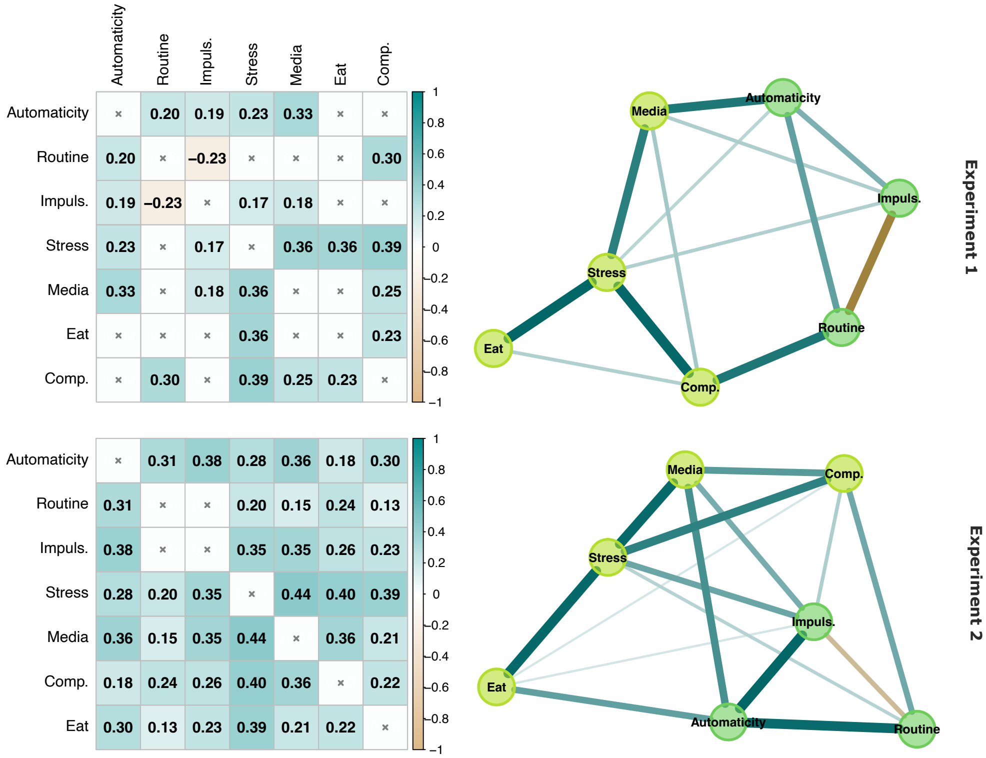

Fig 2. Experiment 1 and 2 networks, using only subscales common to both experiments. Correlation matrices of dimensions used as nodes in the networks (only correlations with $p < 0.05$ are displayed) and networks of relationships between habit and mental health factors. Green edges represent positive connections, yellow edges represent negative connections; thicker edges represent stronger connections. Comp = compulsivity; Eat = problematic eating; Impuls. = impulsivity; Media = problematic media consumption. $N_{experiment1} = 381$, $N_{experiment2} = 285$.

between the two experiments (Table 1).

The networks obtained using the subset of subscales common to both experiments closely resembled the networks obtained using all subscales. The main difference was the emergence of a negative link between impulsivity and routine in the restricted Experiment 2 network.

We estimated the stability of the dynamic network through correlation stability coefficients, which indicated a satisfactory stability for edges ($CS_{r=0.7} = 0.522$) and expected influence ($CS_{r=0.7} = 0.564$).

Table 2. Edge weights and stability indices for Experiment 1 and 2 networks obtained using only subscales common to both experiments.

Edge	Exp.	Weight	Mean	95% CI	prop0
Stress—Problematic eating	1	0.263	0.258	[0.168, 0.350]	0.000
	2	0.257	0.234	[0.118, 0.345]	0.000
Stress—Problematic media	1	0.219	0.221	[0.143, 0.301]	0.000
	2	0.256	0.246	[0.132, 0.354]	0.000
Stress—Compulsivity	1	0.259	0.256	[0.161, 0.348]	0.000
	2	0.215	0.211	[0.106, 0.312]	0.000
Routine—Compulsivity	1	0.233	0.228	[0.132, 0.321]	0.000
	2	0.149	0.141	[0.037, 0.255]	0.033
Automaticity—Problematic media	1	0.231	0.225	[0.136, 0.312]	0.000
	2	0.190	0.179	[0.072, 0.286]	0.001
Automaticity—Problematic eating	1	0.000	0.015	[-0.053, 0.045]	0.663
	2	0.164	0.145	[0.041, 0.251]	0.008
Impulsivity—Routine	1	-0.217	-0.205	[-0.313, -0.098]	0.001
	2	-0.117	-0.130	[-0.259, -0.042]	0.338
Impulsivity—Automaticity	1	0.134	0.129	[0.037, 0.230]	0.030
	2	0.262	0.245	[0.130, 0.360]	0.000
Impulsivitity—Problematic media	1	0.084	0.091	[0.019, 0.183]	0.076
	2	0.140	0.138	[0.033, 0.246]	0.010
Routine—Automaticity	1	0.163	0.158	[0.050, 0.268]	0.013
	2	0.253	0.229	[0.105, 0.354]	0.001

Exp. = experiment; 95% CI = 95% confidence interval reflecting 2.5% and 97.5% quantiles of the bootstrapped sampling distribution of nonzero estimates; prop0 = proportion of times the edge was set to zero.

References

1. Grice JW. Computing and evaluating factor scores. Psychological Methods. 2001;6(4):430. doi:10.1037/1082-989X.6.4.430.
