# Supplementary material for: Differential influence of habit components on compulsive and problematic reward-seeking behavior
Source: PLOS Ment Health. 2025 May 21;2(5):e0000323. doi: 10.1371/journal.pmen.0000323 (PMC12798269; doi:10.1371/journal.pmen.0000323)
Supplement: Attachment — Submitted filename: response_to_reviewers.pdf [file pmen.0000323.s003.pdf]

Response to reviewers

Editor

Thank you for submitting your manuscript to PLOS Mental Health. After careful consideration, we feel that it has merit but does not fully meet PLOS Mental Health's publication criteria as it currently stands. As you will see from the reviewers' comments, the manuscript will benefit from more detailed descriptions of the sample characteristics, more explicit definition of conceptualisation, and justification of analytical approaches. Better links between the two studies would also be appreciated. Therefore, we invite you to submit a revised version of the manuscript that addresses the points raised during the review process.

Authors' response: Thank you for your positive evaluation and constructive feedback on our manuscript. Based on your and Reviewer 1's recommendations, we provided a clearer explanation of the conceptual distinction between automaticity and routine as components of habitual behavior. Additionally, we detailed the characteristics of the samples for Experiments 1 and 2, provided a stronger, clearer justification for our analytical approach, and linked the two experiments together better, as you and Reviewer 2 suggested. We describe the changes that were implemented to address these concerns in detail in our point-by-point response below.

Reviewer 1

In this study, the authors explored the roles of different components of habitual behavior—namely, routine and automaticity—in relation to mental health problems. Using questionnaires completed by 666 participants, they conducted dynamic network analyses that showed routine is associated with compulsivity, while automaticity, in contrast, is linked to problematic media consumption. While previous studies demonstrating habit as a unitary variable is associated with compulsivity (Everitt & Robbins, 2005; Gillian & Robbins, 2014), this conclusion this conclusion opens up a new dimension for the study of habits.

The strength of this paper lies in its clear structure and rigorous application of analytical methods. Although I am not an expert in questionnaire analysis, I found the authors' explanation of their methodology both easy to follow and convincingly thorough. The findings regarding the associations between routine, automaticity, compulsivity, and reward-seeking behaviors were reliably reproduced across two experiments, despite using some different questionnaires. There are inconsistencies between the two experiments, but they are reasonably justified in the discussion section. Therefore, the study is quite complete. The primary weakness of the paper lies in its limited illustration of how the key findings—specifically, the associations between routine, automaticity, compulsivity, and reward-seeking behaviors—advance the current research landscape. The following discussion will focus on how the authors could better articulate the broader contributions of these findings to the research community.

Authors' response: We would like to thank you for your positive and constructive feedback, which was very helpful in guiding us to better articulate the main message of the manuscript. We addressed each of your comments below.

Major issues

R1.1. *One factor that limits the impact of the conclusion is that the authors did not clearly differentiate between routine and automaticity. The current descriptions of these two components, found in lines 45-55, do not provide a clear distinction between them. It wasn't until I referred to the cited work "Habits" by Robbins & Costa (2017) that I grasped their nuance differences. As mentioned in the discussion (lines 444-449), one of the goals of this study is to inspire the development of paradigms for experimentally investigating the observed associations. To achieve this, the authors could benefit from providing more precise descriptions, such as specific behavioral patterns driven by each component or concrete examples from daily life. This clarity would help experimenters better define and operationalize the two components in future research and this can help people better see the study's contribution.*

Authors' response: We fully agree that this a central point and that a clearer description of these concepts would be helpful in better streamlining the study's contribution. We have clarified and developed the distinction between routine and automaticity in the introduction as follows (see pp. 2-3, ll. 46-71):

> "Automatic responses refer to relatively simple behaviors (e.g., eating biscuits) that are automatically initiated upon perception of environmental cues (e.g., a packet of biscuits) and executed until completion (e.g., finishing

off the packet). These instances of simple, purely habitual behavior are captured by the COHS automaticity subscale, which measures behavior that is both habitually initiated and habitually executed—that is, completely controlled by habit (e.g., 'I often find myself finishing off a packet of biscuits just because it is lying there'). As such, it is closely related to typical experimental operationalizations of habit, where habitual behavior is often measured by cue-triggered, short behavioral responses, such as keypresses or food consumption[1]. By comparison, routines correspond to behaviors that may be habitually initiated or habitually executed—that is, partially under goal-directed control[1].This includes chunked action sequences executed with a high degree of automaticity[2,3], but dependent on deliberative control to be initiated as components of multistep goal-directed behavioral responses[1,4]. The COHS routine subscale measures individuals' general propensity to show habitual behavior in their daily life (e.g., 'I tend to like routine'); that is, a tendency or preference for structured habitual action sequences which may nevertheless be directed toward a goal. For instance, always parking one's car or bike in the same place might represent a habitually executed chunk of a goal-directed overarching 'going to the movies' behavioral sequence[1,3]. While closer to many real-life occurrences of complex habitual behavior involving a degree of goal-directed control, this dimension of habit is less frequently studied in experimental paradigms[4]. In fact, habitual responding during contingency degradation has been found to correlate with the automaticity subscale, but not with the routine subscale[5], supporting the idea that the COHS captures a dimension of habitual behavior that is missed by traditional habit tasks. Accordingly, the COHS can be understood to measure a broad range of habitual behaviors that vary in terms of goal-directed involvement."

R1.2. *A second area where the authors could improve is by discussing in greater depth how their conclusions can "help to better understand the role of habit in mental health." For example, in line 388, the authors mention that the strong association between automaticity and problematic media consumption implies a link to addiction. It would be helpful if the authors further explored the relationship between routine and mental disorders associated with compulsivity, such as OCD, as well as the relationship between automaticity and addiction. Expanding on these points could provide valuable insights for improving the diagnosis and understanding of mental health conditions.*

Authors' response: Thank you for this helpful and constructive suggestion. We agree that the interpretation of the links we identified between problematic behaviors and habit components would benefit from being more comprehensively explained to broaden the scope of our findings. As a result, we have significantly developed the discussion to better address this concern (see pp. 15-16, ll. 446-509):

"Across two studies, we showed that both compulsive and problematic reward-seeking behaviors were related to habit, extending existing evidence for the implication of a shared dimension related to habitual processes across these mental health difficulties[6,7]. The studies also showed, however, that two components of habitual behavior were differentially related to mental health problems: routine was associated with compulsivity and social anxiety, while automaticity was associated with problematic media consumption and problematic eating. These findings suggest that the distinctions between automaticity and routine might help better understand how an imbalance between goal-directed and habitual control can give rise to different problematic behaviors.

A possible explanation is that the intervention of habitual processes at different levels of behavior, from its instigation to its performance, can render individuals vulnerable to different problematic behaviors. Automaticity captures the tendency to experience bouts of purely habitually-driven behavior, in which neither behavioral initiation nor execution are under goal-directed control[1]. Routine instead denotes a preference for structure or behavioral regularity, suggesting that habitual processes may be deployed in the context of goal-directed behavior, for instance by facilitating its execution[1].

The tendency to have behavior hijacked by habit, as measured by automaticity, could make individuals particularly prone to engage in reward-seeking behaviors when encountering stimuli which have been associated with rewards. The western, highly digitalized environment in which the present study took place is saturated with cues for both media and food rewards[8,9], presenting countless opportunities for habit to drive behavior toward reward pursuit in individuals with high automaticity, despite goal-directed opposition. These episodes of severe imbalance between goal-directed and habitual control are further evidenced by the symptoms network analysis (Fig. 6), which showed that automaticity was specifically associated with symptoms related to craving and self-control failures such as excessive eating or Internet use, and negatively connected to symptoms related to the implementation of rigid dysfunctional behaviors such as dieting. From this perspective, the association between automaticity and different forms of problematic reward-seeking behavior is congruent with evidence

showing that substance use disorder is specifically associated with automaticity[5,10]. Indeed, problematic media consumption is considered to be an increasingly widespread problematic reward-seeking behavior which shares characteristics with addiction, such as psychological dependence and reduced self-regulation[8,11]. Similarly, the association between automaticity and problematic eating behavior could result from an increased difficulty in resisting cue-induced food consumption. The fact that this association was found in Experiment 2 but not Experiment 1 could be explained by the inclusion of the mYFAS scale—a tool specifically geared toward the identification of addictive-like eating behavior—or by the higher prevalence of problematic eating—as measured with the EAT scale. Further analyses conducted on Experiment 2 data without the mYFAS still showed an association between automaticity and problematic eating (see Fig. 3), thereby suggesting that an explanation in terms of sample levels of problematic eating may be more likely.

The association between routine and compulsivity, conversely, illustrates how a tendency to pursue structure and repetition in daily life could, through an increased reliance on habitual execution, be a contributing factor to the generation of situations in which goal-directed intentions are dysfunctionally diverted by habit. Individuals who frequently engage in rigid behavioral sequences (e.g., always following a set of steps in a specific order before going out) may effectively practice these actions chunks to the point where their execution becomes strongly habitual[12]. This repetition may favor the formation of behavioral loops in which individuals remain stuck, despite goal-directed action selection (e.g., checking that the stove is turned off or that the door is locked with the goal of avoiding harm)[13]. This could explain why routine was found to be most strongly associated with rigid dysfunctional behaviors related to checking and ordering in the symptoms network analysis (Fig. 6). [...] The role of impulsivity in the network also highlights how a predominance of habitual over goal-directed control can interact with other psychological traits, resulting in different behavioral manifestations."

Minor issues

R1.3. *I have noticed that the sample is quite imbalanced in terms of gender, with 506 female participants, 169 male participants, and 6 non-binary participants (line 461). Given that gender is an important factor in understanding mental health, could the authors discuss any potential gender-specific impacts on the two target associations? How might these differences influence the findings related to routine, automaticity, compulsivity, and reward-seeking behaviors?*

Authors' response: Following your suggestion, we have integrated a brief discussion of the gender imbalance as a limitation of the study and elaborated on its potential effects on our findings as follows (see p. 17, ll. 550-557):

> "This study has several limitations [...] our sample was imbalanced in terms of gender, which may limit the generalizability of our findings given that gender plays an important role in mental[14]. The higher proportion of women in our sample may have influenced the prevalence of various mental health difficulties, which may in turn have impacted the resulting network. Gender differences could also exist in the balance between goal-directed and habitual behavior; alternatively, gender may moderate the link between habitual processes and different mental health difficulties. Further research is needed to disentangle these effects."

Summary

R1.4. *Overall, the work is thorough. However, adding more detail about the broader implications of the conclusions could make the paper more appealing to a wider audience, particularly those more interested in the findings than in the methodology.*

Authors' response: Thank you again for your very constructive review that greatly helped us improve the manuscript.

Reviewer 2

In their paper "Differential influence of habit components on compulsive and problematic reward-seeking behavior", the authors examine how habitual behavior relates to compulsive and problematic reward-seeking behavior. This is a well powered study looking for latent factors that can impact common psychiatric symptoms in a range of disorder. I also appreciate that the data and code are publicly available. However, it is important to note that participants were undergraduate students or online study participants, and I am assuming without psychiatric disorders. Therefore, while the study can inform research on transdiagnostic markers of mental health it is primarily a study of individual differences.

Authors' response: Thank you for your constructive review, which has helped us to clarify important methodological and theoretical points. We address each comment in detail below.

R2.1. *Authors claim that they follow a transdiagnostic approach. I see how studying underlying dimensions of problem behaviors is relevant for transdiagnostic research, however, the current study did not include individuals with any diagnoses, so this claim must be toned down. What's more, authors should provide much more details on their sample in terms of reporting certain mental health problems or diagnoses.*

Authors' response: We fully agree that a better characterization of our sample in terms of mental health clarifies the scope of the current study. We also understand how our use of the term "transdiagnostic" might have been misleading and that our study is more accurately described as an investigation of individual differences with a dimensional approach. We took the following steps to address these aspects.

First, when it related to our study, we replaced our use of the term "transdiagnostic" with "dimension" (see p. 3, l. 88 and p. 17, l. 539) to clarify that our approach was focused on common dimensions—rather than diagnoses—associated with problematic behaviors.

Second, we made the link between the adoption of a dimensional approach and the choice of a non-clinical population more explicit, as follows:

> "Following a dimensional perspective, we operated under the assumption that mental health difficulties can be conceptualized as continuous dimensions within the population[6, 15]. Consequently, we recruited a non-clinical population in both experiments." p. 3, ll. 108-111.

> "There were no inclusion or exclusion criteria based on mental health diagnosis." p. 3, l. 116.

> "there were no inclusion or exclusion criteria based on mental health diagnosis." p. 4, ll. 133-134.

Third, we discuss how the fact that the study was conducted on a non-clinical population limits the generalizability of the findings and whether habitual behavior is linked to symptom severity across various diagnostic categories remains to be elucidated. The relevant text in the discussion reads as shown below (p. 16, ll. 540-544):

> "This study has several limitations. First, participants were recruited from a non-clinical population. A growing number of studies taking a dimensional perspective on mental health have identified transdiagnostic factors in community samples (e.g.,[6, 16]); nevertheless, whether the associations we identified in this study between habit and problematic behaviors interact with severity levels remains an open question."

Finally, we reported additional information on the level of mental health difficulties in our samples by using cutoffs for each questionnaire subscales when available as you recommended (see p. 8, ll. 234-251). The corresponding changes in the manuscript are described in more detail in our response to your next comment (R2.2.).

R2.2. *In a similar vein with respect to the measures, how did authors select these specific measures based on the problem behaviors they were interested in? I am familiar with some but not with others. Are they normed in the population they are studying, are there cutoff values for clinical group that would indicate whether someone is in a clinically significant spectrum? These details are currently missing. I would want to see certain cutoff ranges in the histogram plots in order to have a better idea of the sample.*

Authors' response: Thank you for raising this very important point. We selected questionnaires that target our dimensions of interest (problematic reward-seeking, compulsivity, impulsivity, and affective stress) based on two criteria. First, we targeted problematic reward-seeking behaviors that had sufficient variance for the modeling of individual differences in community samples. We therefore based our choices on previous pilot studies showing that certain problematic reward-seeking behaviors such as tobacco, alcohol, and substance consumption did not have sufficient variance in non-clinical, undergraduate samples, while other problematic reward-seeking behaviors such as problematic use of the smartphone or problematic eating had sufficient variance to allow statistical modeling of individual differences. Second, we selected questionnaires that have been validated in French-speaking populations and show good psychometric properties. We explain the rationale for questionnaire selection more clearly in the following sections:

"We selected questionnaires which have been validated in French and which target reward-seeking behaviors, compulsivity, impulsivity, as well as factors related to affective stress. For reward-seeking behaviors, we specifically selected questionnaires targeting behaviors which have shown sufficient variance for the modeling of individual differences in community samples in previous pilot studies (e.g., media consumption, problematic eating)." p. 4, ll. 156, 161.

"To assess problematic reward-seeking behaviors, we reused multiple questionnaires from Experiment 1: the EAT[17], IAT[18], and QABB[19,20]. We added the modified Yale Food Addiction Scale 2.0 (mYFAS 2.0; 11 items[9]) as an additional measure of problematic eating, and replaced the PMPUQ with the newer Smartphone Addiction Scale Short Version (SASSV; 10 items[21]), which we thought might capture current smartphone use better. In order to explore how habit might interact with additional problematic reward-seeking behaviors, we also administered the Gaming Addiction Scale (GAS; 7 items[22]), and the Exercise Dependence Scale Revised (EDS; 21 items[23]). Compulsivity was again assessed with the OCI[24].

To assess mental health problems typically associated with problematic reward-seeking and compulsive behaviors, we administered the PSS[25], the PCLS[26], and the CESD[27] as we did in Experiment 1. To target more specific types of anxiety, we also administered the State-Trait Inventory for Cognitive and Somatic Anxiety (STICSA; 42 items[28]) and the Liebowitz Social Anxiety Scale (LSAS; 24 items[29]). " p. 6, ll. 192-205.

As suggested, we have added cutoff values to the plots showing the distributions of questionnaire scores, which provides a better picture of the levels of mental health difficulties in our samples (see the updated Fig. 1 below).

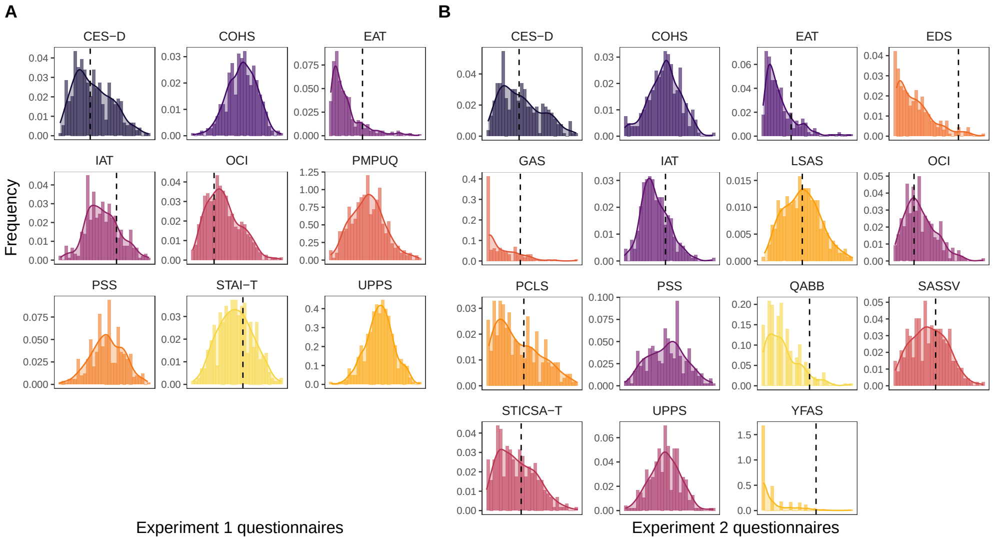

Figure 1. Questionnaire distributions. A: Experiment 1. B: Experiment 2. Dashed lines indicate cutoff scores for the CES-D, EAT, IAT, OCI, STAI-T, LSAS, PCLS, QABB, SASSV, and STICSA; dashed lines indicate what proportion of the sample is categorized as having problematic behaviors for the mYFAS, GAS, and EDS. CESD = Center for Epidemiologic Studies Depression Scale; COHS = Creature of Habit Scale; EAT = Eating Attitudes Test; EDS = Exercise Dependence Scale; GAS = Gaming Addiction Scale; IAT = Internet Addiction Test; OCI = Obsessive-Compulsive Inventory; LSAS = Liebowitz Social Anxiety Scale; PCLS = Post-traumatic Checklist Scale; PMPUQ = Problematic Mobile Phone Use Questionnaire-Short Version; PSS = Perceived Stress Scale; QABB = Questionnaire About Buying Behavior; SASSV = Smartphone Addiction Scale Short Version; STICSA-T = State-Trait Inventory for Cognitive and Somatic Anxiety, trait subscale; STAI-T = State-Trait Anxiety Inventory, trait subscale; UPPS = UPPS Impulsive Behavior Scale; UPPS-P = UPPS-P Impulsive Behavior Scale; mYFAS = modified Yale Food Addiction Scale 2.0.

We explain how the cutoff values were obtained and describe relative levels of mental health difficulties in each sample in the following section (see p. 8, ll. 234-251):

"To characterize the level of mental health difficulties present in each sample, we used cutoff scores when available in the literature. These cutoff values were: 19 for the CES-D,[27], 20 for the EAT[17], 60 for the LSAS[30], 14 for the OCI[31], 44 for the PCLS,[32], 52 for the STAI-T,[33], and 43 for the STICSA-T[34]. Moreover, for the GAS, scores of 'sometimes' or above on at least half the items were taken as indicators of excessive gaming[22]. A score of 50 or above on the IAT indicated frequent problems due to Internet use[18]. For the mYFAS, the recommended scoring procedure was followed to identify food addiction[9]. A score of 10 or above on the QABB was taken to indicate compulsive buying[35]. A score of 32 or above on the SASSV was taken to indicate problematic mobile phone use[21]. The recommended flowchart procedure was followed to identify participants considered to be at risk for exercise dependence[36]. To the best of our knowledge, there are no cutoff scores for the PSS or the UPPS. Fig. 1 shows what proportion of participants is considered to have clinically relevant levels of mental health difficulties, and suggests that this was variable within each sample: the proportion of scores above cutoff values was especially high for questionnaires measuring depression (CES-D), anxiety (STAI-T, LSAS, STICSA-T), obsessive-compulsive behaviors (OCI), and problematic smartphone use (SASSV). Conversely, the proportion of problematic eating (EAT, mYFAS) and problematic exercise was relatively low."

R2.3. *The analyses need to be motivated much more and that goes back to the question of why exactly certain measures were selected in experiment 1 and why the additional instruments were added in experiment 2.*

Authors' response: We agree that the justification for our analytic approach would benefit from being clearer and thank you for raising the need to do so. To respond to your concern, we have elaborated on the reasons leading us to adopt a regularized partial correlation network approach. In short, we used a regularized partial correlation network analysis because this type of analysis allows for capturing and visualizing the complex and interconnected relationships between the multiple variables included in the network, which might be more difficult to assess with more classical analysis such a multiple linear regressions. Partial correlation network models therefore have the potential to provide a more parsimonious and informative representation of the relationships between the different components of habitual behavior and compulsive and problematic reward-seeking behaviors, while also considering the joint influence of other factors such as stress on these relationships. We revised the main text in the materials and methods section (see pp. 8-9, ll. 264-276) to explain this aspect more clearly as detailed in our response to your following comment (R2.4). We have also added more detailed explanations for the choice of questionnaires in Experiment 2, as reported in our response to one of your previous comments (R2.2).

R2.4. *As I understand both EFA and network analyses approaches are highly exploratory and the factor structure and network analyses really depend on the inputs so I am not surprised to see the differences in the factor solutions for experiments 1 and 2. It seems to me that authors had specific hypotheses about how these factors would predict scores on certain questionnaires. Could authors test this with a standard GLM or LME model? I am assuming that the questionnaires also have various items and scales that assess habitual behavior and relationships would reflect this overlap in common measures. So I would want to see with a stepwise approach that latent factors of questionnaire measures, particularly those that are less related to habitual behavior (e.g., media use vs. compulsiveness, which includes having fixed routines) are in fact predicted by habitual behavior.*

Authors' response: Thank you for pointing this out. We understand that our motivations for selecting regularized partial correlation networks over other analytic methods such as hierarchical multiple regression were not clear in the former version of the manuscript. We added a paragraph describing the rationale for choosing this method, which contributed to making the revised manuscript clearer as can be seen in our response to your previous comment (R2.3).

While we did have specific hypotheses regarding the links between the habit components and problematic behaviors, we did not have a specific model for how the other variables (for instance the measures related to stress, depression, and anxiety) would interact with each other and influence these links. Partial correlation networks have the advantage of being able to assess relationships between all variables while controlling for all other variables in the network. While multiple regression can show which independent variables predict a single dependent variable, partial correlation networks can simultaneously assess how independent variables are also predicted by other variables in the network[37]. This allowed us to form a more comprehensive view of how all the variables in our network were linked. In addition, because the LASSO regularization penalizes complexity, this analytic approach accounts for multiple testing[37]. These strengths led us to the determination that regularized partial correlation networks were best suited to test our hypotheses. We agree that this justification for our analytic approach was missing, and since this is a very important point, we expanded on it as indicated below (see pp. 8-9, ll. 264-275):

"We then entered the extracted mental health factors and the personality questionnaires measuring habit components (COHS automaticity and routine) and impulsivity (UPPS and UPPS-P) into a network analysis using a regularized partial correlation network approach. Partial correlation networks model unique interactions between variables, allowing for the assessment of relationships between variables of interest while taking all other variables into account; in other words, partial correlation networks map out linear prediction and multicollinearity among all variables included in the network[37]. Because it penalizes model complexity, regularization allows for model selection to be performed jointly to parameter estimation, resulting in parsimonious networks which are unlikely to contain spurious edges[37]. This analysis therefore allowed us to parsimoniously model and visualize the complex relationships between habit components and compulsive and problematic reward-seeking behaviors, while controlling for the influence of other variables liable to affect these relationships, such as affective stress and impulsivity."

To fully address your concern, we also conducted hierarchical multiple regressions as suggested to further test whether the links we identified with the regularized partial correlations approach between habit components and compulsivity and problematic media use held with a different analytic approach.

These analyses showed that the model which included habit components better predicted problematic media than the model which did not include habit components for Experiment 1 ($\Delta R^2 = 0.056$, $F(2,357) = 18.290$, $p < 0.001$, Table 3) and Experiment 2 ($\Delta R^2 = 0.048$, $F(2,278) = 9.200$, $p < 0.001$, Table 3). Specifically, automaticity significantly predicted problematic media in Experiments 1 and 2 ($\beta = 0.028$, $p < 0.001$ and $\beta = 0.026$, $p < 0.001$ respectively, Table 1), while routine did not ($\beta = 0.002$, $p = 0.731$ and $\beta = 0.002$, $p = 0.880$ respectively, Table 1).

Similarly, the model which included habit components better predicted compulsivity than the model which did not include habit components for Experiment 1 ($\Delta R^2 = 0.069$, $F(2,357) = 16.006$, $p < 0.001$, see Table 4) and Experiment 2 ($\Delta R^2 = 0.027$, $F(2,278) = 4.486$, $p = 0.012$, see Table 4). Specifically, routine significantly predicted compulsivity in Experiments 1 and 2 ($\beta = 0.029$, $p < 0.001$ and $\beta = 0.019$, $p < 0.001$ respectively, Table 2), while automaticity did not ($\beta = -0.007$, $p = 0.244$ and $\beta = -0.011$, $p = 0.131$ respectively, Table 2).

These results are congruent with the results of the network analysis; namely, the habit components are associated with problematic behaviors, with automaticity being selectively associated with problematic media and routine with compulsivity. For the sake of conciseness, we opted not to add these additional analyses in the manuscript. If you believe that these analyses provide an important contribution to the manuscript, we would be happy to include them.

Table 1. Multiple regression results for the prediction of problematic media.

	Experiment 1				Experiment 2			
	Model without habit		Model with habit		Model without habit		Model with habit	
Variable	β(SE)	p-value	β(SE)	p-value	β(SE)	p-value	β(SE)	p-value
Impulsivity	0.139(0.048)	0.906	0.104(0.050)	0.037	0.026(0.007)	< 0.001	0.017(0.001)	0.019
Stress	0.344(0.056)	< 0.001	0.300(0.055)	< 0.001	0.259(0.064)	< 0.001	0.228(0.063)	< 0.001
Prob. eating	-0.114(0.052)	0.031	-0.115(0.051)	0.024	0.038(0.059)	0.515	0.001(0.058)	0.980
Compulsivity	0.134(0.053)	0.013	0.117(0.054)	0.032	0.129(0.056)	0.022	0.129(0.055)	0.020
Automaticity	-	-	0.028(0.006)	< 0.001	-	-	0.026(0.006)	< 0.001
Routine	-	-	0.002(0.006)	0.731	-	-	0.002(0.006)	0.880

Table 2. **Multiple regression results for the prediction of compulsivity.**

	Experiment 1				Experiment 2			
	Model without habit		Model with habit		Model without habit		Model with habit	
Variable	β(SE)	p-value	β(SE)	p-value	β(SE)	p-value	β(SE)	p-value
Impulsivity	-0.114(0.048)	0.018	-0.038(0.049)	0.440	0.007(0.008)	0.353	0.012(0.008)	0.117
Stress	0.307(0.056)	< 0.001	0.300(0.053)	< 0.001	0.216(0.068)	0.002	0.188(0.068)	0.006
Prob. eating	0.082(0.052)	0.112	0.078(0.050)	0.118	0.042(0.062)	0.496	0.048(0.062)	0.439
Prob. media	0.128(0.051)	0.013	0.110(0.051)	0.032	0.145(0.063)	0.022	0.150(0.064)	0.020
Automaticity	-	-	-0.007(0.006)	0.244	-	-	-0.011(0.007)	0.131
Routine	-	-	0.029(0.006)	< 0.001	-	-	0.019(0.006)	< 0.001

Table 3. **Summary of hierarchical multiple regression results for the prediction of problematic media.**

	Experiment 1		Experiment 2	
	Model without habit	Model with habit	Model without habit	Model with habit
R^2	0.179	0.235	0.221	0.269
Adjusted R^2	0.170	0.222	0.209	0.253
ΔF	-	18.290(2,357)	-	9.200(2,278)
p-value	-	< 0.001	-	< 0.001

Table 4. **Summary of hierarchical multiple regression results for the prediction of compulsivity.**

	Experiment 1		Experiment 2	
	Model without habit	Model with habit	Model without habit	Model with habit
R^2	0.168	0.237	0.126	0.153
Adjusted R^2	0.159	0.224	0.113	0.135
ΔF	-	16.006(2,357)	-	4.486(2,278)
p-value	-	< 0.001	-	0.012

R2.5. *I would also want to see a replication of the structure with the initial instrument set from experiment 1 to experiment 2.*

Authors' response: Thank you for this helpful suggestion. We agree that a direct replication of the network obtained in Experiment 1 using only questionnaires common to both experiments is a valuable analysis to assess the robustness of our findings. We have run the analyses you suggested, which show a clear replication of both the EFA structure and the network structure. We reported these findings in the supporting information, the relevant section reads as follows (see pp. 18-22, ll. 602-642):

> "
>
> **Replication**
>
> In order to further estimate the reliability of the links between habit and mental health problems, we ran additional factor and network analyses on the set of questionnaires used in both experiments: the COHS, CES-D, EAT, IAT, OCI, and PSS. The STAI-T and PMPUQ were therefore not included in the analyses for Experiment 1, and the EDS, GAS, LSAS, PCLS, QABB, SASSV, STICSA-T, and mYFAS were not included in the analyses for Experiment 2. Following the same data analysis strategy as described in the main text, we conducted an EFA

followed by a network analysis for Experiments 1 and 2 each, allowing for a direct comparison between the factors and networks identified in each experiment.

Factor analysis

EFAs were performed on the 20 questionnaire subscales common to both Experiment 1 and 2 (Fig. 2). The correlations between subscales were sufficiently large for an EFA (Experiment 1: $X^2_{(190)} = 3774.764$, $p < .001$, Experiment 2: $X^2_{(190)} = 1934.540$, $p < .001$). The factor analyses converged toward two very similar four-factor solutions (Fig. 2).

The validity coefficients (Experiment 1: $R^2 = 0.966, 0.950, 0.963, 0.877$; Experiment 2: $R^2 = 0.965, 0.954, 0.900, 0.997$) assessing the potential impact of factor score indeterminacy[38] were satisfactory, allowing factor scores derived from the EFAs to be used in network analyses.

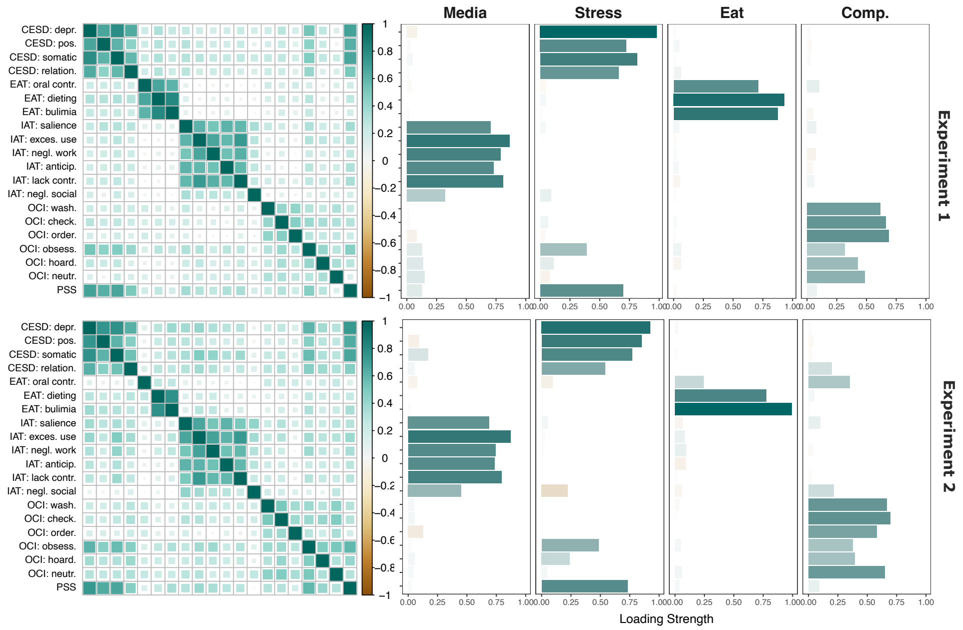

Figure 2. Correlation matrix and standardized loadings for Experiment 1 and 2, using only subscales common to both experiments. Standardized loadings for questionnaire subscales into each factor are displayed in green for positive values and yellow for negative values. CESD = Center for Epidemiologic Studies Depression Scale; EAT = Eating Attitudes Test; IAT = Internet Addiction Test; OCI = Obsessive-Compulsive Inventory Revised; PSS = Perceived Stress Scale; depr. = depressive affect; pos. = positive mood; relation. = disturbed interpersonal relationships; contr. = control; exces. use = excessive use; negl. = neglect; anticip. = anticipation; wash. = washing; check. = checking; order. = ordering; obsess. = obsessing; hoard. = hoarding; neutr. = neutralizing; danger. = dangerous use; prohib. = prohibity use; depend. = dependence. $N_{experiment1} = 381$, $N_{experiment1} = 285$.

Network analysis

Fig. 3 illustrates the dynamic networks we used to estimate the connections between the two components of habitual behavior, impulsivity, and the EFA-extracted mental health factors.

The resulting networks had strong similarities (see 3): routine and automaticity were positively related, automaticity was associated with problematic media while routine was associated with compulsivity, impulsivity was positively associated with automaticity and negatively associated with routine, and affective stress was associated with all problematic behavior nodes.

The main difference between the two networks in terms of links between nodes was the positive association between automaticity and problematic eating in Experiment 2, which was not found in Experiment 1. Interestingly, while the overall network structures were similar, the centrality of each node within the network was variable between the two experiments (see Table 5).

The networks obtained using the subset of subscales common to both experiments closely resembled the networks obtained using all subscales. The main difference was the emergence of a negative link between

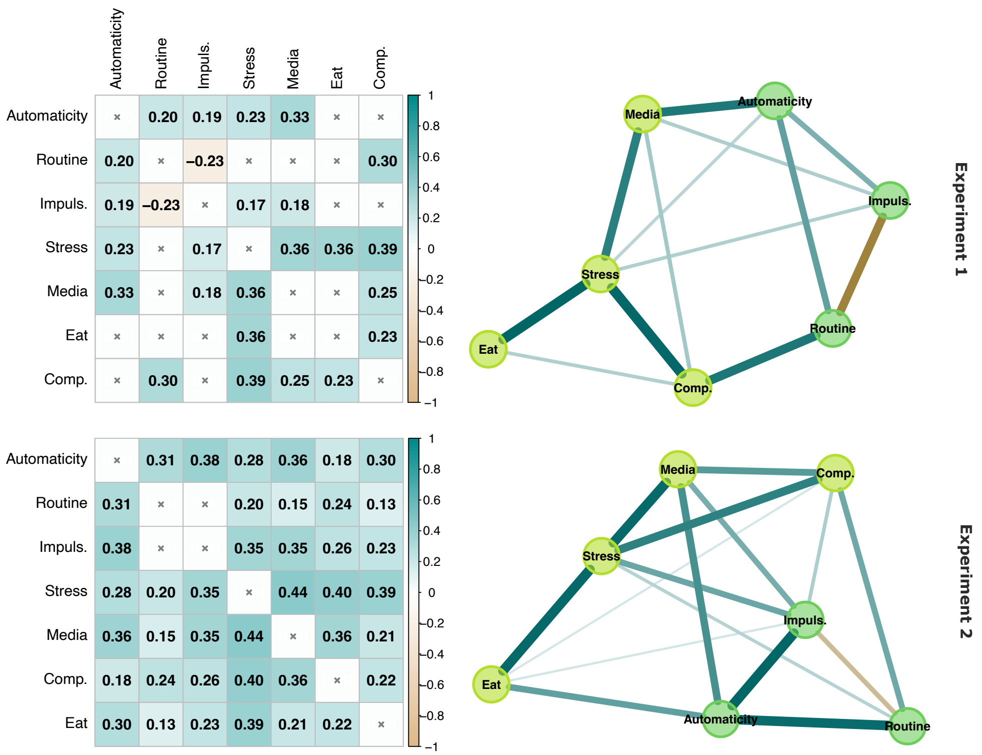

Figure 3. Experiment 1 and 2 networks, using only subscales common to both experiments. Correlation matrices of dimensions used as nodes in the networks (only correlations with $p > 0.05$ are displayed) and networks of relationships between habit and mental health factors. Green edges represent positive connections, yellow edges represent negative connections; thicker edges represent stronger connections. Comp = compulsivity; Eat = problematic eating; Impuls. = impulsivity; Media = problematic media consumption. $N_{experiment1} = 381$, $N_{experiment2} = 285$.

Table 5. Expected influence of the graphical LASSO network nodes, using only subscales common to Experiments 1 and 2.

	Experiment 1	Experiment 2
Automaticity	0.396	0.962
Routine	-1.049	-1.476
Problematic media	0.494	0.431
Stress	1.400	1.370
Problematic eating	-0.485	-0.768
Compulsivity	0.626	-0.024
Impulsivity	-1.382	-0.493

impulsivity and routine in the restricted Experiment 2 network.

We estimated the stability of the dynamic network through correlation stability coefficients, which indicated a satisfactory stability for edges ($CS_{r=0.7} = 0.522$) and expected influence ($CS_{r=0.7} = 0.564$). " ll. 601-644

Table 6. **Edge weights and stability indices for Experiment 1 and 2 networks obtained using only subscales common to both experiments.**

Edge	Exp.	Weight	Mean	95% CI	prop0
Stress—Problematic eating	1	0.263	0.258	[0.168, 0.350]	0.000
	2	0.257	0.234	[0.118, 0.345]	0.000
Stress—Problematic media	1	0.219	0.221	[0.143, 0.301]	0.000
	2	0.256	0.246	[0.132, 0.354]	0.000
Stress—Compulsivity	1	0.259	0.256	[0.161, 0.348]	0.000
	2	0.215	0.211	[0.106, 0.312]	0.000
Routine—Compulsivity	1	0.233	0.228	[0.132, 0.321]	0.000
	2	0.149	0.141	[0.037, 0.255]	0.033
Automaticity—Problematic media	1	0.231	0.225	[0.136, 0.312]	0.000
	2	0.190	0.179	[0.072, 0.286]	0.001
Automaticity—Problematic eating	1	0.000	0.015	[-0.053, 0.045]	0.663
	2	0.164	0.145	[0.041, 0.251]	0.008
Impulsivity—Routine	1	-0.217	-0.205	[-0.313, -0.098]	0.001
	2	-0.117	-0.130	[-0.259, -0.042]	0.338
Impulsivity—Automaticity	1	0.134	0.129	[0.037, 0.230]	0.030
	2	0.262	0.245	[0.130, 0.360]	0.000
Impulsivitity—Problematic media	1	0.084	0.091	[0.019, 0.183]	0.076
	2	0.140	0.138	[0.033, 0.246]	0.010
Routine—Automaticity	1	0.163	0.158	[0.050, 0.268]	0.013
	2	0.253	0.229	[0.105, 0.354]	0.001

Exp. = experiment; 95% CI = 95% confidence interval reflecting 2.5% and 97.5% quantiles of the bootstrapped sampling distribution of nonzero estimates; prop0 = proportion of times the edge was set to zero.

R2.6. *Finally, the paper is hard to read in this form because figures and figure captions are separated and in methods and results, analyses are not sufficiently motivated. It would be important to improve clarity by motivating the analyses more and tying them back to the hypotheses.*

Authors' response: We apologize for this formatting issue. To improve the readability of the manuscript, we have added a version of the manuscript that includes all figures and their captions embedded in the main text. We also clarified the links between the hypotheses and the analyses. The revised passages now read as follows:

> "To do so, we adopted a dimensional approach to mental health[15], which conceptualizes mental health problems as dynamic systems interacting within and across diagnostic categories[39].We used factor analysis to extract mental health dimensions from questionnaires assessing various problematic behaviors. We then implemented network analyses in order to characterize the links between two components of habitual behavior and dimensions of problematic reward-seeking and compulsive behaviors." p. 3, ll. 88-92.

> "For each experiment,we first applied an exploratory factor analysis (EFA) to the mental health questionnaire subscalesto extract common dimensions from this mental health space. We then used network analysis to test whether the two components of habitual behavior were associated with problematic reward-seeking and compulsive behaviors. To further assess the links between habit and specific mental health difficulties, we conducted an additional network analysis at the symptom level on data pooled from Experiments 1 and 2." pp. 7-8, ll. 221-227.

"This analysis therefore allowed us to parsimoniously model and visualize the complex relationships between habit components and compulsive and problematic reward-seeking behaviors, while controlling for the influence of other variables liable to affect these relationships, such as affective stress and impulsivity." pp. 8-9, ll. 271-275.

We hope to have satisfactorily addressed the motivation for our analyses in our responses to your comments R2.2., R2.3. and R2.4. Thank you again for your constructive feedback, which has allowed us to strengthen our manuscript in several aspects.

References

1. Robbins, T. W. & Costa, R. M. Habits. *Curr. Biol.* **27**, R1200–R1206, DOI: 10.1016/j.cub.2017.09.060 (2017).
2. Dezfouli, A. & Balleine, B. W. Habits, action sequences and reinforcement learning. *Eur. J. Neurosci.* **35**, 1036–1051, DOI: 10.1111/j.1460-9568.2012.08050.x (2012).
3. Ersche, K. D., Lim, T.-V., Ward, L. H., Robbins, T. W. & Stochl, J. Creature of habit: A self-report measure of habitual routines and automatic tendencies in everyday life. *Pers. Individ. Differ.* **116**, 73–85, DOI: 10.1016/j.paid.2017.04.024 (2017).
4. Gardner, B., Rebar, A. L., de Wit, S. & Lally, P. What is habit and how can it be used to change real-world behaviour? narrowing the theory-reality gap. *Soc. Pers. Psychol. Compass* **18**, e12975 (2024).
5. Ersche, K. D. *et al.* Reduced glutamate turnover in the putamen is linked with automatic habits in human cocaine addiction. *Biol. Psychiatry* **89**, 970–979, DOI: 10.1016/j.biopsych.2020.12.009 (2021).
6. Gillan, C. M., Robbins, T. W., Sahakian, B. J., van den Heuvel, O. A. & van Wingen, G. The role of habit in compulsivity. *Eur. Neuropsychopharmacol.* **26**, 828–840, DOI: 10.1016/j.euroneuro.2015.12.033 (2016).
7. Voon, V. *et al.* Disorders of compulsivity: A common bias towards learning habits. *Mol. Psychiatry* **20**, 345–352, DOI: 10.1038/mp.2014.44 (2015).
8. Cataldo, I., Billieux, J., Esposito, G. & Corazza, O. Assessing problematic use of social media: where do we stand and what can be improved? *Curr. Opin. Behav. Sci.* **45**, 101145, DOI: 10.1016/j.cobeha.2022.101145 (2022).
9. Brunault, P. *et al.* The modified yale food addiction scale 2.0: validation among non-clinical and clinical french-speaking samples and comparison with the full yale food addiction scale 2.0. *Front. Psychiatry* **11**, 480671 (2020).
10. Wyckmans, F. *et al.* Habitual routines and automatic tendencies differential roles in alcohol misuse among undergraduates. *Front. Psychol.* **11**, 607866, DOI: 10.3389/fpsyg.2020.607866 (2020).
11. Sun, Y. & Zhang, Y. A review of theories and models applied in studies of social media addiction and implications for future research. *Addict. Behav.* **114**, 106699, DOI: 10.1016/j.addbeh.2020.106699 (2021).
12. Fradkin, I., Adams, R. A., Parr, T., Roiser, J. P. & Huppert, J. D. Searching for an anchor in an unpredictable world: A computational model of obsessive compulsive disorder. *Psychol. Rev.* **127**, 672–699, DOI: 10.1037/rev0000188 (2020).
13. Robbins, T. W., Vaghi, M. M. & Banca, P. Obsessive-compulsive disorder: puzzles and prospects. *Neuron* **102**, 27–47, DOI: 10.1016/j.neuron.2019.01.046 (2019).
14. Hill, T. D. & Needham, B. L. Rethinking gender and mental health: A critical analysis of three propositions. *Soc. Sci. & Medicine* **92**, 83–91, DOI: 10.1016/j.socscimed.2013.05.025 (2013).
15. Dalgleish, T., Black, M., Johnston, D. & Bevan, A. Transdiagnostic approaches to mental health problems: Current status and future directions. *J. Consult. Clin. Psychol.* **88**, 179, DOI: 10.1037/ccp0000482 (2020).
16. Seow, T. X. F. & Gillan, C. M. Transdiagnostic Phenotyping Reveals a Host of Metacognitive Deficits Implicated in Compulsivity. *Sci. Reports* **10**, 2883, DOI: 10.1038/s41598-020-59646-4 (2020).
17. Leichner, P., Steiger, H., Puentes-Neuman, G., Perreault, M. & Gottheil, N. Validation of an eating attitude scale in a French-speaking Quebec population. *Can. J. Psychiatry. Revue Can. de Psychiatr.* **39**, 49–54, DOI: 10.1177/070674379403900110 (1994).
18. Khazaal, Y. *et al.* French validation of the internet addiction test. *Cyberpsychology & Behav.* **11**, 703–706, DOI: 10.1089/cpb.2007.0249 (2008).
19. Lejoyeux, M., Tassain, V., Solomon, J. & Ades, J. Study of compulsive buying in depressed patients. *J. Clin. Psychiatry* **58**, 169–173 (1997).
20. Billieux, J., Rochat, L., Rebetez, M. M. L. & Van der Linden, M. Are all facets of impulsivity related to self-reported compulsive buying behavior? *Pers. Individ. Differ.* **44**, 1432–1442, DOI: 10.1016/j.paid.2007.12.011 (2008).
21. Lopez-Fernandez, O. Short version of the smartphone addiction scale adapted to spanish and french: Towards a cross-cultural research in problematic mobile phone use. *Addict. behaviors* **64**, 275–280 (2017).
22. Khazaal, Y. *et al.* Psychometric properties of the 7-item game addiction scale among french and german speaking adults. *BMC Psychiatry* **16**, 1–10 (2016).
23. Kern, L. Validation de l'adaptation française de l'échelle de dépendance à l'exercice physique: l'eds-r. *Pratiques Psychol.* **13**, 425–441 (2007).

24. Zermatten, A., Van der Linden, M., Jermann, F. & Ceschi, G. Validation of a French version of the Obsessive–Compulsive Inventory-Revised in a non-clinical sample. *Eur. Rev. Appl. Psychol.* **56**, 151–155, DOI: 10.1016/j.erap.2005.07.003 (2006).

25. Lesage, F.-X., Berjot, S. & Deschamps, F. Psychometric properties of the French versions of the Perceived Stress Scale. *Int. J. Occup. Medicine Environ. Heal.* **25**, 178–184, DOI: 10.2478/S13382-012-0024-8 (2012).

26. Yao, S.-N. *et al.* Évaluation des états de stress post-traumatique: Validation d'une échelle, la PCLS. *L'Encéphale: Revue de Psychiatr. Clinique Biol. et Thérapeutique* (2003).

27. Morin, A. J. *et al.* Psychometric properties of the Center for Epidemiologic Studies Depression Scale (CES-D) in French clinical and nonclinical adults. *Revue d'Epidemiologie et de Santé Publique* **59**, 327–340, DOI: 10.1016/j.respe.2011.03.061 (2011).

28. Ree, M. J., French, D., MacLeod, C. & Locke, V. Distinguishing cognitive and somatic dimensions of state and trait anxiety: Development and validation of the state-trait inventory for cognitive and somatic anxiety (sticsa). *Behav. Cogn. Psychother.* **36**, 313–332 (2008).

29. Yao, S.-N. *et al.* L'anxiété sociale chez les phobiques sociaux: Valiation de l'échelle d'anxiété sociale de liebowitz (version francaise). *L'Encéphale: Revue de psychiatrie clinique biologique et thérapeutique* (1999).

30. Caballo, V. E., Salazar, I. C., Arias, V., Hofmann, S. G. & Curtiss, J. Psychometric properties of the Liebowitz Social Anxiety Scale in a large cross-cultural Spanish and Portuguese speaking sample. *Braz. J. Psychiatry* **41**, 122–130, DOI: 10.1590/1516-4446-2018-0006 (2018).

31. Abramowitz, J. S. & Deacon, B. J. Psychometric properties and construct validity of the Obsessive–Compulsive Inventory—Revised: Replication and extension with a clinical sample. *J. Anxiety Disord.* **20**, 1016–1035, DOI: 10.1016/j.janxdis.2006.03.001 (2006).

32. Blanchard, E. B., Jones-Alexander, J., Buckley, T. C. & Forneris, C. A. Psychometric properties of the PTSD checklist (PCL). *Behav. Res. Ther.* **34**, 669–673, DOI: 10.1016/0005-7967(96)00033-2 (1996).

33. Wiglusz, M. S., Landowski, J. & Cubała, W. J. Psychometric properties and diagnostic utility of the State–Trait Anxiety Inventory in epilepsy with and without comorbid anxiety disorder. *Epilepsy & Behav.* **92**, 221–225, DOI: 10.1016/j.yebeh.2019.01.005 (2019).

34. Van Dam, N. T., Gros, D. F., Earleywine, M. & Antony, M. M. Establishing a trait anxiety threshold that signals likelihood of anxiety disorders. *Anxiety, Stress. & Coping* **26**, 70–86, DOI: 10.1080/10615806.2011.631525 (2013).

35. Lejoyeux, M., Mathieu, K., Embouazza, H., Huet, F. & Lequen, V. Prevalence of compulsive buying among customers of a Parisian general store. *Compr. Psychiatry* **48**, 42–46, DOI: 10.1016/j.comppsych.2006.05.005 (2007).

36. Hausenblas, H. A. & Downs, D. S. How Much is Too Much? The Development and Validation of the Exercise Dependence Scale. *Psychol. & Heal.* **17**, 387–404, DOI: 10.1080/0887044022000004894 (2002).

37. Epskamp, S. & Fried, E. I. A tutorial on regularized partial correlation networks. *Psychol. Methods* **23**, 617, DOI: 10.1037/met0000167 (2018).

38. Grice, J. W. Computing and evaluating factor scores. *Psychol. Methods* **6**, 430, DOI: 10.1037/1082-989X.6.4.430 (2001).

39. Fried, E. I. *et al.* Mental disorders as networks of problems: a review of recent insights. *Soc. Psychiatry Psychiatr. Epidemiol.* **52**, 1–10 (2017).
