# Supplementary material for: Differential influence of habit components on compulsive and problematic reward-seeking behavior
Source: PLOS Ment Health. 2025 May 21;2(5):e0000323. doi: 10.1371/journal.pmen.0000323 (PMC12798269; doi:10.1371/journal.pmen.0000323)
Supplement: Attachment — Submitted filename: response_to_reviewers_auresp_2.pdf [file pmen.0000323.s004.pdf]

Response to reviewers

Editor

Thank you for submitting your manuscript to PLOS Mental Health. After careful consideration, we feel that it has merit but does yet not fully meet PLOS Mental Health's publication criteria as it currently stands. Therefore, we invite you to submit a revised version of the manuscript that addresses the points raised during the review process, especially on self-reported diagnoses (if any) or discuss its potential limitation to the current study.

Authors' response: Thank you for your constructive feedback on our manuscript. Based on your and Reviewer 2's recommendation, we discussed how the absence of self-report diagnostic information constitutes a limitation to the current study. We describe the changes that were implemented to address this concern in our response below.

Reviewer 1

I am generally satisfied with the revisions made and the improvements to the manuscript. It is evident that the authors have put considerable effort into clarifying the distinction between routine and automaticity, as well as expanding the implications of their findings. I believe these revisions greatly enhance both the clarity and the contribution of the study.
The authors' expanded explanation in the introduction now provides a much clearer differentiation between routine and automaticity. The inclusion of real-life examples and descriptions significantly improves the conceptual distinction between these two components, and I believe this will help readers better understand the study's contribution.
Additionally, the authors have made a strong effort to expand the discussion on the broader implications of their findings. The added details regarding how these habitual components may contribute to mental health conditions such as substance use disorder, and problematic eating provide valuable context and will be helpful for researchers in clinical psychology and related fields.
I have no further concerns regarding the publication of this manuscript in its current form.

Authors' response: We would like to thank your again for your very valuable feedback and for your positive evaluation of our work.

Reviewer 2

Authors have much improved the manuscript in their revision. An issue that remains is that authors do not report any mental health self report questions in their sample. If this study was done with a clinical focus in mind, I would think that asking subjects to self report diagnoses, treatments including medication would be important. This is particularly important given that a significant proportion of participants self-report anxiety and OCD symptoms in the clinically significant range. If this has not been assessed and or information is not available to authors, this should be clearly stated as a limitation of the current study

Authors' response: Thank you for your constructive feedback and for your positive evaluation of our revised manuscript. We unfortunately did not assess self-reported information concerning mental health diagnoses and treatment, and we agree that this constitutes a limitation to this study. As recommended, we now clearly report this in the participants section, acknowledge this limitation, and discuss its implications, as follows:

> "There were no inclusion or exclusion criteria based on mental health diagnosis, and participants were not asked to report whether they had received a formal diagnosis or whether they were undergoing treatment for mental health difficulties." p. 3, ll. 111-113.

> "In addition, we did not ask participants whether they had received a mental health diagnosis, or whether they were undergoing any type of treatment for mental health difficulties. We therefore do not know whether the relatively high proportion of clinically relevant levels of depression, anxiety, obsessive-compulsive behaviors, and mobile phone use was associated with formal diagnoses and treatment, and whether this impacted our findings." p. 15, ll. 550-555.
